# Manual Linear Movements to Assess Spasticity in a Clinical Setting

**DOI:** 10.1371/journal.pone.0053627

**Published:** 2013-01-15

**Authors:** Lucio Marinelli, Carlo Trompetto, Laura Mori, Gabriele Vigo, Elisabetta Traverso, Federica Colombano, Giovanni Abbruzzese

**Affiliations:** Clinica Neurologica, Department of Neurosciences, Rehabilitation, Ophthalmology, Genetics, Maternal and Child Health, University of Genova, Genova, Italy; Weill Cornell Medical College, United States of America

## Abstract

In a clinical setting, where motor-driven systems are not readily available, the major difficulty in the assessment of the stretch reflex lies in the control of passive limb displacement velocity. A potential approach to this problem arises from the use of manual sinusoidal movements (made by continuous alternating flexions and extensions) paced by an external stimulus. Unfortunately, there are conditions in which sinusoidal movements induce interfering phenomena such as the shortening reaction or postactivation depression. In the present paper, a novel manual method to control the velocity of passive linear movements is described and the results obtained from both healthy subjects and spastic patients are reported. This method is based on the synchronisation of movements with tones played by a metronome at different speeds. In a first set of experiments performed in healthy subjects, we demonstrated consistent control of velocity during passive limb movements using this method. Four joints usually examined during muscle tone assessment were tested: wrist, elbow, knee and ankle joints. Following this, we conducted a longitudinal assessment of the stretch reflex amplitude in wrist flexor muscles in patients with spasticity treated with botulinum toxin type A. The evaluators were not only able to vary the movement velocity based on the metronome speed, but also could reproduce the respective speeds two weeks later, despite the changing degree of hypertonia. This method is easy to perform in a clinical setting and hardware requirements are minimal, making it an attractive and robust procedure for the widespread clinical assessment of reflex hypertonia.

## Introduction

Studies utilizing motor-driven systems that are capable of producing passive limb displacements at predetermined velocities have clearly shown that increased excitability of the stretch reflex (SR) is a primary effector of spasticity [1] and rigidity [2], the two most common forms of hypertonia. In SR assessment, the velocity of motor-driven displacements is by definition pivotal in spasticity; likewise, it is also an important factor when the SR is used in the evaluation of rigidity [2]. At the same time, compelling evidence points to the altered mechanical properties of muscles and joints secondary to immobilization as another distinct determinant of hypertonia in spastic and rigid patients [3]. This contribution is more prevalent in patients evaluated during advanced clinical stages. To distinguish between these mechanisms, hypertonia has been divided into two components: SR mediated hypertonia (reflex hypertonia) and hypertonia due to changes in muscle and joint properties (non-reflex hypertonia).

Movements executed by motor-driven devices to elicit the SR can be either sinusoidal or “ramp and hold” displacements (herein referred as “linear movements”). Sinusoidal displacements consist of rhythmic and consecutive flexion and extension movements, while linear movements involve displacement of a limb from one position to another and stopping without a consequent movement.

In a clinical setting where motor-driven systems are not readily available, hypertonia is commonly evaluated by means of subjective clinical scales incapable of differentiating between the reflex and non-reflex components [4]–[6]. This results in an inaccurate basis for the selection of treatment modality, many of which can vary in efficacy depending on the predominant component of hypertonia [7]. Therefore, it is necessary to have a system that can reliably assess the SR in clinical practice.

Without the aid of motor-driven systems, the major difficulty in SR assessment lies in the control of passive limb displacement velocity. A potential approach to this problem arises from the use of manual sinusoidal movements paced by an external stimulus. The evaluator can take advantage of these sinusoidal movements to displace the joint at consistent and accurate velocities, indicating a possible clinical strategy for SR evaluation [6]. Unfortunately, sinusoidal movements are inherently flawed for the comprehensive assessment of the SR. First, they cannot be used for the investigation of the static phase of the SR, which can be present in both spastic and rigid patients [8], [9]. Second, during sinusoidal movements the presence of EMG activity from the shortening muscle (shortening reaction) can obscure the measurement of the SR latency [10], which is an important parameter in the evaluation of spasticity [11]. Third, the continuous sinusoidal movements could reduce the SR amplitude in some spastic patients in a phenomenon called postactivation depression [12]. Therefore, there are conditions in which sinusoidal movements would not be the best choice in the SR assessment and linear movements are preferable as an alternative.

In the present paper, a novel manual method to control the velocity of passive linear movements is described and the results obtained from both healthy subjects and spastic patients are reported. This method is based on the synchronisation of the movements with tones played by a metronome at different speeds. In a first set of experiments performed in healthy subjects, we demonstrated consistent control of velocity during passive limb movements using this method. Four joints usually examined during muscle tone assessment were tested: wrist, elbow, knee and ankle joints. In a second set of experiments performed in patients with spasticity, the validity of this method was demonstrated in a longitudinal SR assessment performed in wrist flexor muscles before and after the injection of botulinum toxin type A (BoNT-A). Part of these findings have been presented at the 6^th^ World Congress of Neurorehabilitation [13].

## Materials and Methods

### Subjects

The first set of experiments was performed on 6 right-handed healthy subjects [two women, age: 30±3 years (mean±SD), range: 26–34 years].

The second set of experiments was performed on 12 patients [two women, age 66±9 (mean±SD), range 48–78 years] with post-stroke hemiparesis who were selected according to the following criteria: (1) clinical presentation of a hemispheric stroke leading to unilateral motor deficit at least 9 months prior to participation; (2) CT or MRI documenting a single vascular lesion in the middle cerebral artery territory; (3) a stable clinical picture in the last three months; (4) presence of spasticity in the wrist flexor muscles with a Modified Ashworth Scale (MAS) score ranging from 1 to 3 (1, 1+, 2, 3); (5) no previous treatment with BoNT-A. All subjects gave informed consent according to the Declaration of Helsinki. The participants provided their written informed consent to participate in this study, which was notified to the local ethical committee “Comitato Etico IRCCS Azienda Ospedaliera Universitaria San Martino – IST”, according to the “Determinazione AIFA 20 Marzo 2008” (http://www.agenziafarmaco.gov.it/sites/default/files/det_20marzo2008.pdf). Demographic and clinical details of the 12 patients are reported in Table 1.

**Table 1:** Clinic and demographic features of patients.

Subject	Age/sex	Lesioned hemisphere	BoNT-A dosage	Baseline MAS	Test MAS
1	75 M	L	30U	1+	0
2	64 M	R	70U	2	2
3	68 M	R	50U	2	1
4	48 M	R	70U	3	2
5	63 F	R	50U	2	1+
6	65 M	L	50U	2	2
7	67 M	L	40U	2	0
8	78 M	L	75U	3	1+
9	71 M	R	30U	1	1
10	56 M	R	40U	1+	1
11	61 F	L	50U	2	1
12	75 M	R	75U	3	2

### Evaluators

Passive movements on the six healthy subjects were performed by six evaluators [two male medical doctors, two female physiotherapists and two medical students (one woman); age: 33±10 years (mean±SD), range: 23–50 years], one for each tested subject.

Passive movements on the 12 stroke patients were performed by two evaluators [two medical doctors (one woman); age: 30–35 years], herein referred to as evaluator A and evaluator B. Both evaluators tested all 12 patients. Therefore, each patient was tested twice.

To ensure a standardized level of training, all evaluators were properly instructed in a 15–20 minute training session.

### Instrumentation

For the kinematic recording of the passive movements and the assessment of the passive range of movement (ROM), we used two independent methods: the optoelectronic system “ELITE” (BTS S.p.a., Milan, Italy) and the Biopac MP100 data acquisition system connected to a TSD130B twin-axis electronic goniometer (Biopac Systems Inc, USA).

The ELITE system consists of six infrared cameras (100 Hz sampling rate) that track the motion of passive reflective markers in 3D coordinates. Synchronised acquisition and data processing was performed using Analyser software (BTS S.p.a.). Three reflective markers (15 mm in diameter) were positioned for each investigated joint: the first on the proximal segment, the second near the joint pivot and the third on the distal segment.

The goniometer was placed across the investigated joint in order to optimally record the angle during the joint displacements; a sampling rate of 2 KHz was used.

EMG activity was recorded by surface electrodes (TSD150B, Biopac Systems Inc, USA) placed over the muscle belly. The signal was acquired by a MP100 unit (Biopac Systems Inc, USA) with a 2 KHz sampling rate and underwent a Blackman −61dB 80–300 Hz band-pass filter for offline processing (AcqKnowledge 3.8.1 software by Biopac Systems Inc, USA). A constant-current stimulator (model DS7A, Digitimer, UK) was used to stimulate the median nerve.

Movement timing was paced with a software emulated metronome.

### The Method: from Sinusoidal to Linear Movements with the Help of Motor Imagery

The method consisted of 4 phases. During all of the phases, the subject was instructed to stay relaxed and to avoid resisting or facilitating the joint movements applied by the evaluator. Upper limb joints were assessed with the subject in a supine position while lower limb joints were evaluated with the subject in a prone position, with the feet off the surface of the examination table.

#### Phase 1: setting the frequency

An audio tone frequency was set on a metronome. For instance, the frequency of 60 beats per minute (BPM) was set. This means that the interval between two consecutive tones (tone interval, TI) corresponded to 1000 ms. Only the evaluator could perceive the tones through earphones.

#### Phase 2: following the rhythm

While trying to cover the entire ROM, the evaluator applied continuous manual sinusoidal joint displacements, moving the distal segment of the joint smoothly. These movements were done at a constant pace so that the distal segment of joint would arrive at the extreme flexed and extended positions in synchrony with consecutive tones. In this way, the evaluator related the motion with the rhythm of the metronome.

#### Phase 3: from overt movement to motor imagery

When the evaluator had comfortably synchronized the movement with the tones, the sinusoidal movement was stopped at one extreme position (flexed or extended). At this point, the evaluator continued to conceptualize the previously executed movement using first perspective motor imagery [14] with the metronome tone as a cue.

#### Phase 4: from motor imagery to overt movement

**Figure 1:**
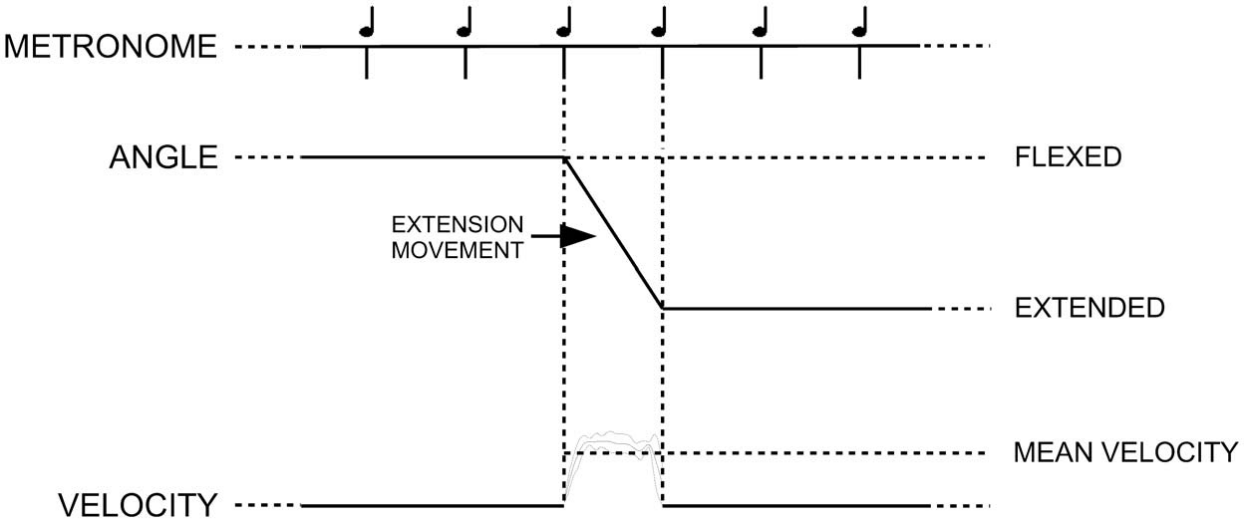
Experimental procedure. During phase 4 the evaluator performs a smooth extension movement which starts and ends in synchrony with the metronome tones. The mean velocity is derived from the resulting velocity profile.

After at least 15 seconds of motor imagery, when the evaluator felt prepared to follow the pace of the metronome accurately, he moved the distal segment of the joint so that it arrived at the opposite position in synchrony with the following tone; then he stopped again (Fig. 1).

Through this procedure, linear movement was derived from sinusoidal movement while retaining the feature of velocity control. This was made possible by the utilization of both an external audio cue as well as first person motor imagery. This procedure was repeated several times, in order to collect several flexion and extension linear movements.

### Experimental Procedure in Healthy Subjects

The method was applied to wrist, elbow, knee and ankle joints all examined on the right side. Before the application of the method, the passive ROM of the tested joint was evaluated. In the assessment of wrist, elbow and knee joints, the metronome was set at the following BPM values: 40BPM (TI = 1500 ms), 60BPM (TI = 1000 ms), 120BPM (TI = 500 ms) and 180BPM (TI = 330 ms). Since the ankle is the joint with the smallest ROM, we used the BPM values of 60BPM, 120BPM, 180BPM and 240BPM (TI = 250 ms) in order to achieve joint velocities comparable with the other three joints. At each BPM value, 15 flexion and 15 extension linear movements were collected. Flexion was considered the movement reducing the angle between skeletal members.

### Experimental Procedure in Stroke Patients

The method was applied to the wrist joint of the affected side before (baseline condition) and 15 days after (test condition) the injection of BoNT-A in the *flexor carpi radialis* muscle (FCR). The metronome was set at the following BPM values: 40 BPM (TI = 1500 ms) and 60 BPM (TI = 1000 ms). At each BPM value, 15 extension and 15 flexion linear movements were collected. Only the extension movements were analysed. As in healthy subjects, just before the application of the method, passive ROM of the wrist joint was evaluated. During the application of the method, EMG activity was recorded from FCR, which was stretched by the passive extension linear movements.

The M-wave was recorded in the FCR through supramaximal electrical stimulation of the median nerve in the cubital fossa.

Wrist flexor muscle hypertonus was clinically rated at baseline condition and test condition according to the MAS. BoNT-A was dosed from 30 to 75 Xeomin® Units, according to the clinically appropriate dose (Table 1).

### Kinematic Data Analysis

Passive ROM evaluated before the application of the method was assessed as the difference between joint angles at the maximal flexed and extended positions.

For each linear movement obtained in phase 4, onset and termination times were calculated. With the ELITE system, these times were identified on the velocity profile, considering the 1% of the peak velocity amplitude as threshold. With the electronic goniometer, they were visually detected, using a display gain of 20°/1cm and a temporal window of 340 ms/1cm.

Onset and termination times were used to calculate the following two parameters: 1) ***dynamic-ROM*** calculated as the difference between joint angles at the starting and ending positions; 2) movement mean velocity (**mean-V**), calculated as *dynamic*-ROM/movement duration (difference between onset and termination times). ***Dynamic*** **-ROM** is specified in order to differentiate it from the ROM measured before the application of the method in a static condition.

Since a preliminary analysis showed no difference between the kinematic parameters acquired using the optoelectronic system and the electronic goniometer, only the latter are shown in the results.

### EMG Data Analysis

The SR amplitude was measured as the mean amplitude of the rectified EMG during the passive displacements. The SR amplitude was divided by the amplitude of the M-wave elicited at baseline condition to obtain SR/M ratio.

### Statistical Analysis

In the normal subject group, the comparison between ROM and *dynamic*-ROM was performed using a factorial ANOVA, while the 4 BPM have been compared using repeated measures ANOVA for each joint separately and with BPM as within-subjects factor. Linear regression analysis was performed to determine the correlation between BPM and mean-V.

In the patients groups *dynamic*-ROM, mean-V and SR/M have been compared before and after the BoNT-A treatment using a repeated-measures ANOVA, with the variable “condition” (baseline and test) as within-subjects factor and the variables “BPM” (40BPM and 60BPM) and “evaluator” (A and B) as between-subjects factors. Baseline and test ROM were compared using a paired T-test.

All analyses were considered significant for p<0.05. All the measures of variability are expressed as standard deviation.

## Results

### Healthy Subjects

Table 2 shows ROM and *dynamic*-ROM values for all the tested joints. In each joint, *dynamic*-ROM was lower than ROM (wrist: F(1,20) = 213, p<0.0001; elbow: F(1,20) = 80, p<0.0001; knee: F(1,20) = 38, p<0.0001; ankle: F(1,20) = 134, p<0.0001), without any difference among BPM (wrist: F(3,20) = 0.72, p = 0.55; elbow: F(3,20) = 0.57, p = 0.64; knee: F(3,20) = 0.48, p = 0.70; ankle: F(3,20) = 2.0, p = 0.15). The value of *dynamic*-ROM, expressed as a percentage of ROM, was 82±6% for the wrist, 96±6% for the elbow, 86±13% for the knee and 79±9% for the ankle. No significant difference was found between baseline and test ROM (p = 0.2).

**Table 2:** Range of movement in healthy subjects.

	ROM	*dynamic*ROM	*dynamic*ROM	*dynamic*ROM	*dynamic*ROM	*dynamic*ROM
		*40 BPM*	*60 BPM*	*120 BPM*	*180BPM*	*240 BPM*
**WRIST**	141±4	119±10	113±12	112±6	119±7	
**ELBOW**	135±8	127±7	127±7	133±9	136±7	
**KNEE**	127±9	107±17	105±17	107±19	118±15	
**ANKLE**	47±3		35±4	35±4	37±5	41±3

**Figure 2:**
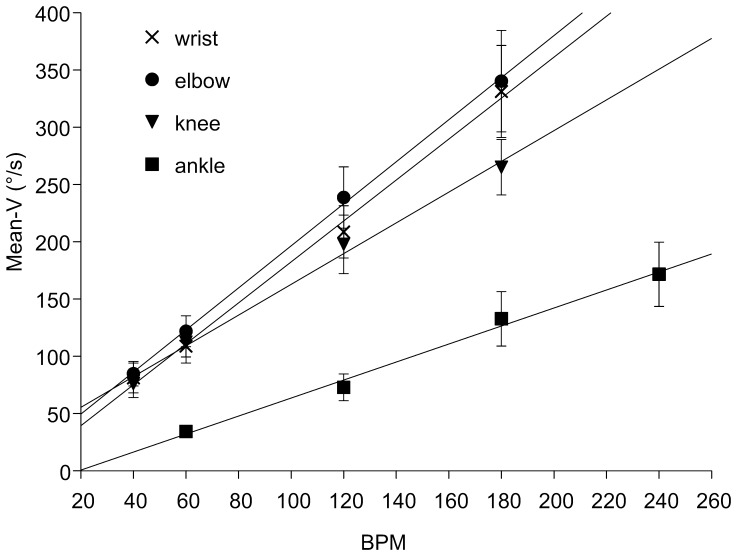
Mean-V in the normal subjects group has a direct linear relationship with BPM for all the tested joints according to the following functions. Wrist: y = 1.8*x+3.8, elbow: y = 1.8*x+1.3, knee: y = 1.3*x+29.5, ankle: y = 7.9*x–15.1.

Mean-V has a direct linear relationship with BPM for all the tested joints (Fig. 2). The fit functions are reported in the figure legend, the squared-r values were 0.99 for all the 4 correlations. Since the ankle ROM was smaller than the ROM of the other joints, the mean-V values obtained from the movement of the ankle were consequently lower, despite the use of higher BPM values.

### Stroke Patients

Table 3 shows ROM and *dynamic*-ROM values for the wrist joint obtained by evaluator A and B, at baseline and test conditions. The value of *dynamic*-ROM, expressed as a percentage of ROM, was 90±21% during baseline and 87±15% during test conditions. *Dynamic*-ROM was lower than ROM both at baseline (F [1,44] = 31, p<0.0001) and test conditions (F [1,44] = 156, p<0.0001). *Dynamic*-ROM was similar between baseline and test conditions (F [1,44] = 1.3, p = 0.3). No significant difference was found between BPM (F [1,44] = 0.003, p = 1) nor was it found between evaluators (F [1,44] = 0.01, p = 0.9). No significant interactions CONDITION X BPM (F(1,44) = 0.04, p = 0.9) and CONDITION X EVALUATORS (F(1,44) = 0.008, p = 0.9) were found.

**Table 3:** Range of movement in patients.

	ROM	*dynamic-*ROM		*dynamic-*ROM	
		*40 BPM*		*60 BPM*	
		A	B	A	B
**Baseline**	125±17	110±25	113±24	113±23	111±33
**Test**	133±16	115±17	117±20	116±19	114±21

**Figure 3:**
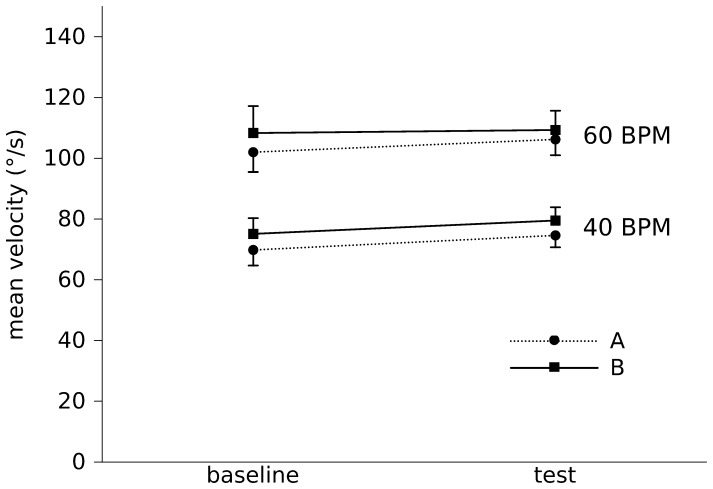
Mean-V in the patients group is plotted in baseline and test conditions. The filled circles connected with dashed line represent the evaluator A, while the filled squares connected with a solid line represent the evaluator B. Mean-V is higher at 60 BPM without any difference between baseline and test conditions or between evaluator A and B.

Figure 3 shows that mean-V at 60BPM was higher than mean-V at 40BPM (F [1,44] = 41, p<0.0001). No significant difference was found between baseline and test conditions (F [1,44] = 1.3, p = 0.3) nor was it found between operators (F [1,44] = 1.0, p = 0.3). No significant interactions CONDITION X BPM (F [1,44] = 0.1, p = 0.8) and CONDITION X EVALUATORS (F [1,44] = 0.08, p = 0.8) were found. Considering both evaluators and conditions, the average mean-V at 60BPM was 106±24°/s and at 40BPM 75±16°/s.

**Figure 4:**
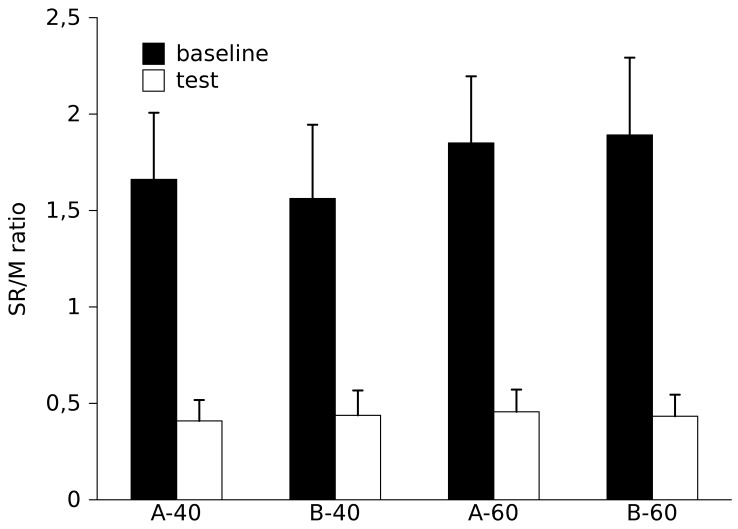
The SR/M ratio is higher at baseline compared to the test condition. The trend toward an increased SR/M at 60 BPM does not reach significance. No difference can be found between the two evaluators.

In all the patients, passive wrist extension movements evoked a SR on the FCR. No EMG activity was recorded while the muscle was in the shortened position before the passive extension. SR/M ratios were higher during baseline condition compared to the test condition (F [1,44] = 93.1, p<0.0001). No difference was found between evaluators (F [1,44] = 0.03, p = 0.9) and between BPM (F [1,44] = 1.2, p = 0.3) (Fig. 4). No significant interactions CONDITION X BPM (F [1,44] = 1.4, p = 0.2) and CONDITION X EVALUATORS (F [1,44] = 0.0003, p = 1) were found.

Table 1 shows MAS scores of the 12 patients at baseline and test conditions. While in 3 patients MAS scores did not change, in the other 9 patients MAS scores were reduced following the injection of BoNT-A.

## Discussion

### Healthy Subjects

For all the joints, the *dynamic*-ROM was smaller than ROM. It should be considered that during ROM assessment, evaluators had sufficient time to reach the extreme joint positions, minimizing the risk of damaging the joint. However, during the application of the method when *dynamic*-ROM was assessed, the evaluators were likely aware that the kinetic energy applied to the distal segment could have damaged the joint. The evaluators’ consideration to preserve the joint probably limited the passive movement, thus explaining the reduction of *dynamic*-ROM in comparison to ROM.

The most important finding was that the mean-V increased linearly with progressive BPM values for all the tested joints. Furthermore, the gap between the *dynamic*-ROM and ROM was not a factor in this linear relationship as the difference was consistent across the tested BPM frequencies.

The range of BPM was large enough to produce mean-V values from about 30 to 400°/s, covering all the angular velocities used in a clinical setting [1], [2]. The linear relationship between mean-V and BPM values in the six healthy subjects was plotted using the data obtained by six different evaluators who all received a brief training session. This indicates that the method is easy to use and produces reproducible results across a variety of evaluators.

These results prompted us to investigate this method’s efficacy in assessing the SR in patients with spasticity.

### Kinematic Results in Stroke Patients

*Dynamic*-ROM was smaller than ROM without any difference among evaluators, BPM values, and between conditions. Theoretically, the SR in wrist flexor muscles could have played a major role in this discrepancy. During ROM assessment, the evaluators stretched the wrist flexor muscles at speeds below the SR threshold, whereas when the *dynamic*-ROM was measured a SR was evoked in these muscles. The presence of the SR may have limited extension movements, thereby reducing *dynamic-*ROM. However, we assert that this is not the case for the following reasons. First, a similar difference between the *dynamic-*ROM and ROM was also found in healthy subjects, in whom a SR was not evoked. Second, the difference did not change after BoNT-A injection, when the SR amplitude was decreased. Therefore, we believe that the most likely explanation for the discrepancy between *dynamic-*ROM and ROM is that the evaluators were concerned for the integrity of the joint, as was suggested for the healthy subjects. However another possible explanation can also be proposed. When the examiner performs the linear movements, he is very concentrated in keeping them synchronized with the metronome while he is less concerned about reaching the actual extreme joint position. Also in normal subjects, just before reaching the extreme position when moving a joint a mild resistance related to the fibro-elastic properties of the joint and muscles con be perceived. In this sort of “dual task” condition, the role of the mild resistance near the end of the movement might disguise the examiner in considering to have reached the extreme position when he starts perceiving this mild resistance, resulting in an overall reduced dynROM.

In both the baseline and test conditions, the mean-V obtained at 60 BPM was higher than that obtained at 40 BPM. Furthermore, there was no difference in mean-V values between the two evaluators as well as between the two conditions. Therefore, the passive movements obtained with our method demonstrated all the features necessary for a longitudinal SR assessment.

In the case that the treatment with BoNT-A had induced an increase of the *dynamic*-ROM, the mean-V obtained at test condition would have been higher than that obtained at baseline condition. This was not the case in the present set of experiments, as the treatment did not increase the *dynamic*-ROM. This can be attributed to the fact that we only enrolled patients who did not have important ROM limitations of the wrist joint before BoNT-A injection.

Even if the treatment had resulted in an increased ROM, two strategies would have been used to achieve comparable velocities between baseline and test conditions. The first strategy would have been to predetermine and limit the joint displacement instead of moving throughout the entire ROM. The second strategy would have consisted of decreasing the BPM value during the test condition. Further studies performed in patients with severe spasticity and ROM limitations are needed to test these procedures.

### EMG Results in Stroke Patients

A SR was evoked in each patient during the baseline condition, reflecting the presence of reflex hypertonia in all 12 tested subjects. The SR amplitude was highly variable among the 12 patients as has been reported in the literature [15]. This high variability is explained by the fact that this parameter depends heavily on factors such as the muscle size, skin resistance, thickness of the subcutaneous tissue, among many others. EMG normalisation was therefore needed in order to compare the SR amplitude among subjects. This point was crucial, as it has recently been shown that normalisation is essential to correctly interpret the changes following BoNT-A treatment [16].

To reduce the inter-subject variability, we divided the SR amplitude by the M-wave amplitude obtaining the SR/M ratio [17]. Since M-wave amplitudes are expected to be reduced after BoNT-A injection [18], we used the M-wave values obtained at baseline condition to normalise the SR amplitudes elicited before and after the treatment.

SR/M ratios did not differ between the two evaluators. Furthermore, SR/M ratios did not differ between the two BPM values. Based on the findings in healthy subjects, we decided to use 40 and 60 BPM values to test the accuracy of our method in producing passive displacements at different velocities. We can now say that a gap of 500 ms between the two tested TIs (40BPM = TI 1500; 60BPM = TI 1000) yielded two appreciably distinct movement velocities. As the difference between the two velocities was small (31°/s), we did not find any difference between the SR/M ratios obtained at the two BPM values. This is consistent with previous results obtained from motor-driven systems [1].

For both the evaluators, the SR/M ratios decreased after BoNT-A treatment. In each patient, the SR/M ratio at test condition was less the 50% of the ratio at baseline condition. Again, these results are corroborated by previous findings obtained with motor-driven systems [19].

### Conclusion

The method was consistent at achieving discrete velocities during linear passive movements in both healthy subjects and stroke patients. In the latter, the method was useful in detecting the reflex component of hypertonia as well as the effects induced by BoNT-A treatment.

The method is easy to perform in a clinical setting and the hardware requirements are minimal, making it an attractive and robust procedure for widespread clinical assessment of reflex hypertonia.
